# WGM Single‐Molecule Validation of pH‐Dependent Molecular Energetics at Gold Nanointerfaces: Influence of Buffer Molecules on Glyphosate Ligand Binding

**DOI:** 10.1002/advs.77567

**Published:** 2026-09-14

**Authors:** Kulathunga Mudalige Kalani Perera, Srikanth Pedireddy, Sivaraman Subramanian, Frank Vollmer

**Affiliations:** ^1^ Department of Physics and Astronomy Living Systems Institute University of Exeter Exeter UK

**Keywords:** binding site, buffer molecule, chemistry, density functional theory, ionic bonding, ligand, nanoparticle, nanorod, oxyanion, physisorption, WGM

## Abstract

Single‐molecule sensors are increasingly applied to small multifunctional molecules whose nanoparticle interactions are governed by multiple functional groups and ionic states. At this scale, ligand and buffer molecules become chemically comparable, raising a fundamental question: how does a small multifunctional ligand compete with buffer molecules for nanoparticle binding sites when both carry similar reactive groups? We address this question using whispering gallery mode (WGM) optoplasmonic sensors, which resolve individual glyphosate (GLP) binding events at gold nanorods in real time and without labels. We show that glyphosate interactions shift from permanent cooperative binding at alkaline pH, where amine and oxyanion groups outcompete carbonate for gold surface sites, to transient physisorption mediated by surface‐adsorbed buffer molecules at lower pH. For the first time, a label‐free single‐molecule technique consolidates these observed kinetics with complexation energies, binding geometries, and coordination motifs predicted by density functional theory (DFT) and confirmed by surface‐ enhanced Raman spectroscopy (SERS), establishing a general framework for probing competitive ligand binding at nanointerfaces.

## Graphical abstract

Single‐molecule optical sensing reveals how pH and buffer molecules regulate glyphosate interactions with gold nanorods. Combined experiments and theoretical calculations show that changes in molecular charge and competition from buffer species determine whether glyphosate binds transiently or persistently. The findings highlight buffer composition as an important consideration when designing and interpreting nanoscale sensing experiments.

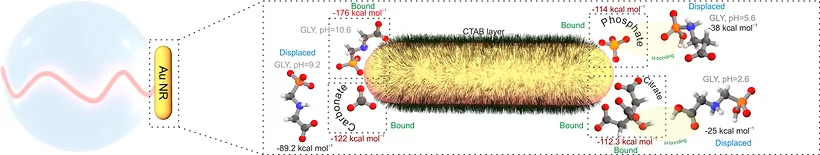

## 1 Introduction

Molecular interactions at nanostructured interfaces underpin critical processes in catalysis, sensing, environmental chemistry, and nanomedicine [1, 2]. At such interfaces, the binding configuration, energetics, and dynamics of individual molecules determine nanoparticle (NP) stability, surface reactivity, and functional performance. Achieving a comprehensive understanding of these interactions, particularly their dynamics across different buffer environments, remains a major challenge. This challenge is especially critical for small, multifunctional molecules whose adsorption behaviour depends sensitively on pH, ionic strength, and competition from buffer species prevalent in real environments.

Gold nanoparticles (AuNPs) provide a well‐established model system for investigating molecular interactions owing to their chemical stability, tuneable plasmonic properties, and versatile surface coordination chemistry [3, 4]. Extensive research has elucidated the binding mechanisms of AuNPs with diverse ligands, such as thiols, amines, and oxyanions [4, 5, 6]. Thiol ligands, for example, form strong Au─S bonds that yield well‐ordered monolayers capable of significantly modifying surface chemistry and catalytic activity. In contrast, amines and oxyanions interact through complex mechanisms involving electrostatic forces, hydrogen bonding, and coordination chemistry [7, 8, 9, 10]. Many environmentally and biologically relevant molecules, however, contain multiple functional groups capable of interacting simultaneously with the nanoparticle surface, leading to pH‐dependent, multidentate, and dynamic binding configurations [10, 11, 12, 13, 14]. For such systems, adsorption is inherently dynamic and highly sensitive to surrounding solution chemistry, yet the molecular mechanisms governing these processes remain incompletely understood.

Experimental insights into NP–molecule interactions have traditionally relied on ensemble‐averaged techniques such as localized surface plasmon resonance (LSPR) spectroscopy, surface‐enhanced Raman spectroscopy (SERS), and fluorescence‐based assays [11, 15, 16, 17, 18, 19, 20]. However, these techniques exhibit inherent limitations in capturing transient and dynamic binding events with high temporal and molecular resolution [11, 15, 16, 17, 18, 19, 20]. Weak, short‐lived interactions often escape detection due to rapid dissociation kinetics, while strong binding events require single‐event resolution that remains challenging when only a few molecules interact per nanoparticle. Moreover, fluorescence‐based single‐molecule approaches typically rely on chemical labelling, which alters the intrinsic physicochemical properties of the analyte and limits applicability to specific molecular systems. These limitations emphasize the critical need for a label‐free sensing platform with high sensitivity and molecular specificity, capable of preserving native molecular behavior while resolving complex interaction dynamics at the NP interface in real time.

In this work, we address this gap by employing an optoplasmonic sensing platform that integrates optical microcavities as WGM resonators with AuNRs [21, 22], enabling real‐time, label‐free detection of molecular interactions at the single‐molecule level [23, 24]. Using this platform, we investigate glyphosate (GLP), an organophosphorus herbicide of environmental and health significance. GLP exhibits multiple pH‐dependent protonation states arising from its carboxyl, phosphonate, and amine functional groups, resulting in variable binding affinities at the nanoparticle interface [25, 26, 27]. By combining single‐molecule WGM sensing with density functional theory (DFT) simulations, we directly resolve the real‐time binding kinetics and thermodynamic parameters influencing GLP binding on AuNRs. Our findings establish a DFT‐based framework for predicting interaction energies of competing ligands and validate these predictions against experimental data. Our results reveal that buffer molecules can significantly influence the ligand‐binding kinetics at the AuNR surface, uncovering molecular mechanisms that are often overlooked in single‐molecule sensing, particularly for multifunctional molecules such as GLP. This combined approach provides mechanistic insight into pH‐regulated nanointerfacial chemistry and establishes a general approach for predicting molecular binding behavior at nanoparticle surfaces under realistic conditions, thereby informing the design of next‐generation nanoparticle systems for environmental monitoring and molecular recognition.

## 2 Results and Discussion

### 2.1 Correlating WGM, SERS, and DFT to Resolve pH‐Dependent Binding Energetics

**FIGURE 1:**
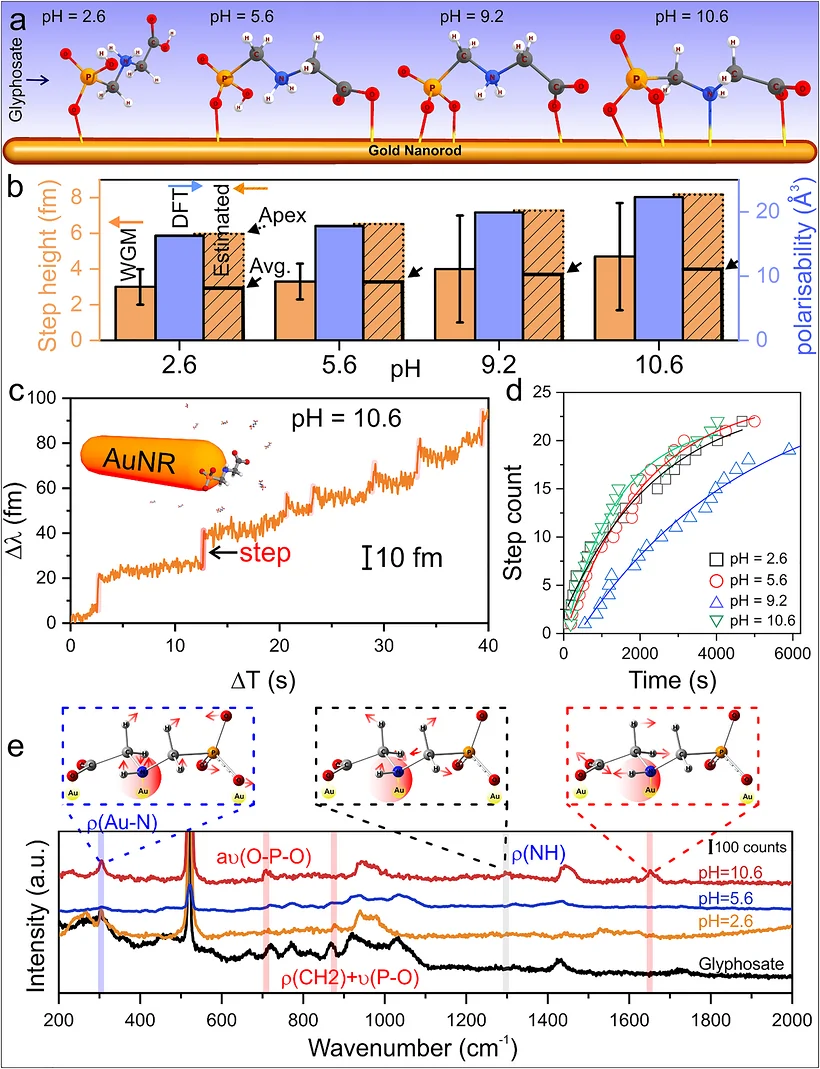
pH‐dependent GLP–AuNR interactions investigated by WGM sensing, DFT, and SERS. (a) Schematic representation of GLP binding configurations at the AuNR surface at pH 2.6, 5.6, 9.2, and 10.6. (b) Experimentally measured WGM step heights (Δλ), DFT‐calculated molecular polarisabilities (α), and predicted WGM step heights for GLP under the investigated pH conditions. Error bars represent standard deviations from 25 binding events per condition. (c) Representative WGM resonance time trace showing step‐like Δλ shifts for GLP–AuNR interactions at pH 10.6. (d) Cumulative binding‐event counts as a function of time at pH 2.6, 5.6, 9.2, and 10.6, with fitted curves. (e) SERS spectra of GLP at pH 2.6, 5.6, and 10.6, with selected vibrational modes indicated. Experiments were conducted at room temperature.

**FIGURE 2:**
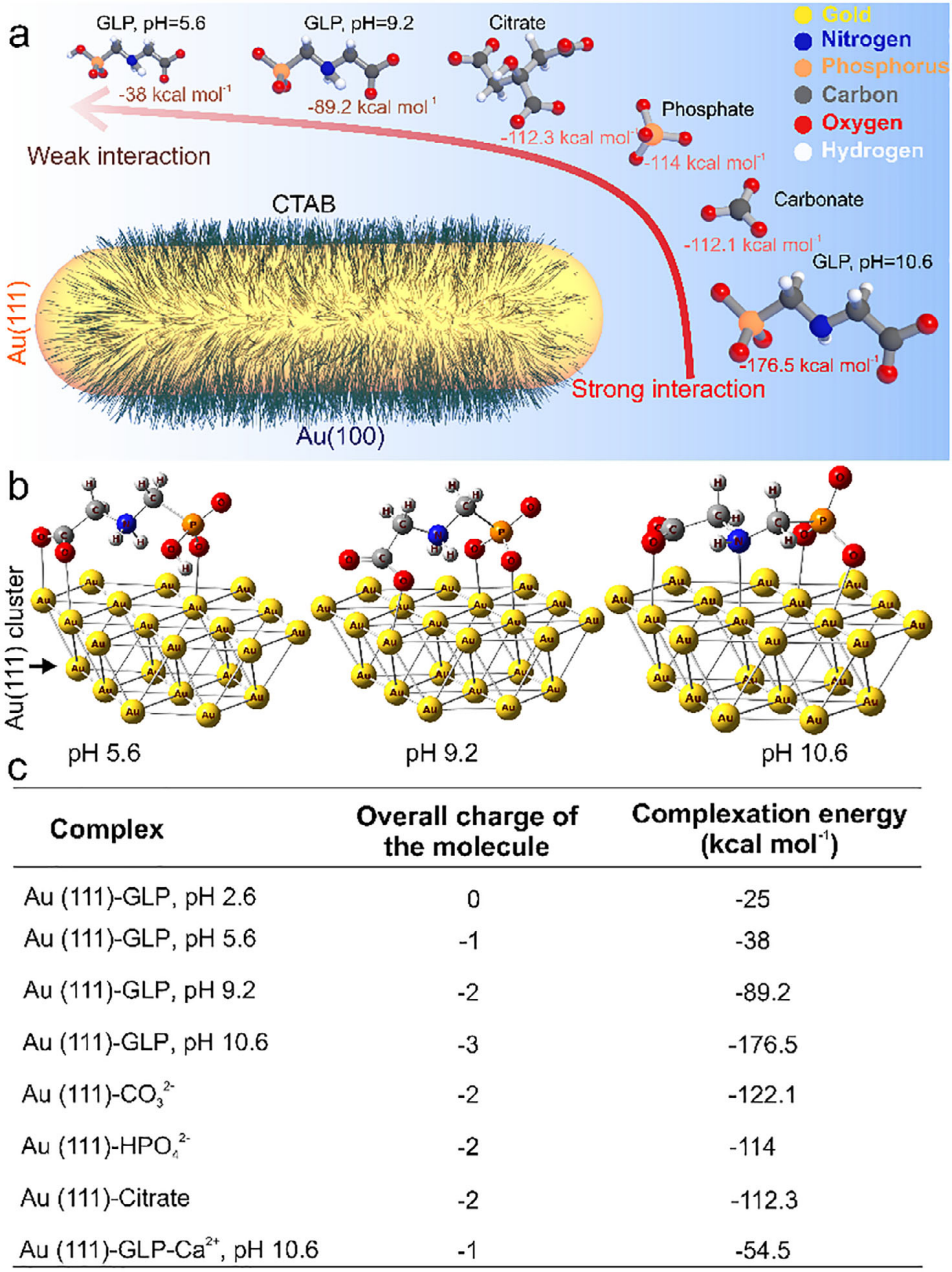
DFT analysis of pH‐dependent GLP interactions with Au(111). (a) Schematic representation of GLP and buffer species interacting with the AuNR surface, with DFT‐calculated complexation energies (kcal mol^−^^1^). (b) DFT‐optimised structures of GLP on the Au(111) surface at pH 5.6, 9.2, and 10.6. (c) Summary of the molecular charges and DFT‐calculated complexation energies of GLP and buffer species interacting with Au(111).

To rationalise the experimentally observed pH‐dependent binding behavior at the atomic scale, DFT calculations are employed in three complementary modalities to interpret the single‐molecule WGM measurements. First, DFT identifies the molecular binding geometries of GLP on the Au surface (Figure 1a), providing molecular‐level insight into how this multifunctional ligand interacts with gold as a function of pH. Second, the calculations quantify the pH‐dependent molecular polarisability of GLP (Figure 1b and Table S1), which governs the magnitude of the WGM resonance shift arising from single‐molecule interactions. Third, DFT evaluates the GLP–Au interaction energies (Figure 2), providing a thermodynamic framework for interpreting the observed single‐molecule binding kinetics. The calculations show that GLP binding is governed by the protonation‐dependent availability of its phosphonate, carboxylate, and amine functional groups, which interact with the gold surface through distinct coordination modes under different pH conditions (Figure 1a). These pH‐dependent ionic states therefore provide a key experimental parameter for controlling and tuning GLP–Au interactions.

To isolate the intrinsic pH‐dependent GLP–Au binding behavior, solution pH is adjusted using HCl/NaOH in the presence of 20 mM NaCl as the background electrolyte, without the addition of buffer salts [28]. This approach avoids the intentional introduction of competing buffer anions, leaving Cl^−^ and OH^−^ as the only anionic species present in solution. CTAB‐functionalised AuNRs were employed throughout, with dense CTAB coverage passivating the nanorod side facets while leaving the tips accessible for GLP adsorption (Figure S2) [6, 14]. The stability of the CTAB‐coated AuNRs under the investigated pH and buffer conditions was confirmed by complementary zeta‐potential, UV–vis, and visual stability measurements, while stable WGM resonances and reproducible binding responses further supported the integrity of the immobilized AuNR sensing platform throughout the experiments (Figure S4). WGM sensing detects single‐molecule binding as discrete resonance wavelength shift (Δλ, in femtometres), which are proportional to the molecule's excess polarisability (see SI for details) [23, 29, 30, 31]. Following GLP addition, discrete step‐like resonance shifts (1–20 fm) are observed at all pH values investigated (Figure 1c, Figure S5), consistent with individual binding events at exposed AuNR sites. The variation in step heights can be attributed to the spatially non‐uniform field intensity around the gold nanorod tips (Figure S8), which leads to position‐dependent contributions of individual binding events to the overall resonance shift. Statistical analysis of the WGM step‐height distributions, including nonparametric hypothesis testing and violin‐plot visualization, confirms a significant overall pH dependence of the single‐molecule responses, whereas the distinction between the pH 9.2 and pH 10.6 binding regimes is primarily determined by binding kinetics rather than step amplitude alone (Figure S9).

Across the full pH range, the experimentally measured average WGM step heights Δλ upon GLP binding show good quantitative agreement with DFT‐calculated molecular polarisabilities of GLP in its adsorption configuration at the gold interface, (Figure 1b, for details on DFT calculations and averaging see SI section of Figure S8 and Table S1). The agreement between the WGM sensing results and DFT predictions (Figure 1b, Table S1) demonstrates that the WGM sensor responds to ionic‐state‐dependent variations in the effective molecular polarisability of GLP at the single‐molecule level, providing direct experimental validation of the DFT‐based calculations.

The pH dependence of GLP–Au binding is further reflected in the cumulative step‐count kinetics (Figure 1d). At pH 10.6, where the amine group is deprotonated and available for Au coordination, the step count rises most steeply and saturates (∼22 events) within ∼2000 s, consistent with the strongest DFT‐predicted binding energy (−176 kcal mol^−^^1^) arising from cooperative multi‐site coordination via the carboxylate, phosphonate oxyanion, and amine lone pair. At pH 2.6, 5.6, and 9.2, where the amine remains protonated, saturation is reached over a longer timescale (∼3000–6000 s), reflecting weaker phosphonate/carboxylate dominated‐adsorption in the absence of an amine contribution.

Complementary surface‐enhanced Raman spectroscopy (SERS) measurements were performed to corroborate the molecular identity and coordination motifs inferred from single‐molecule WGM sensing. DFT‐assisted spectral analysis enables reliable assignment of vibrational features associated with specific functional groups and adsorption geometries (Figures 2, S6 and S7). At pH 10.6, SERS spectra (Figure 1e) are dominated by modes corresponding to carboxylate (COO^−^), phosphonate (PO_3_^2^^−^), and amine (:NH) groups. A prominent band at 306 cm^−^^1^ is assigned to Au–N stretching, providing direct evidence for amine‐mediated coordination, while features at 710 and 1651 cm^−^^1^ confirm involvement of phosphonate and carboxylate groups, respectively. Concurrently, a mode at 1298 cm^−^^1^ corresponds to the in‐plane bending (ρNH) of the NH group, further confirming amine interaction with the Au substrate. As the pH decreases to 5.6, protonation suppresses amine participation and phosphonate–Au interactions become more pronounced, reflected by modes characteristic of HPO_3_^−^ species (e.g., 306, 872, and 1316 cm^−^^1^). The peak at 306 cm^−1^ corresponds to the out‐of‐plane bending mode (ωHO‐P‐O) of phosphate (HPO_3_^−^) interacting with the Au surface. Additional modes at 872 and 1316 cm^−1^ are attributed to the out‐of‐plane bending of HPO_3_^−^ and NH_3_^+^, respectively, consistent with protonation‐driven conformational changes. At pH 2.6, the spectra are dominated by phosphate‐derived vibrations (303 cm^−^^1^ and 900–1050 cm^−^^1^), indicating that adsorption is largely restricted to protonated phosphonate groups under acidic conditions (Figures 1e and S7). These pH‐dependent spectral shifts highlight the dynamic interplay between GLP's functional groups and the Au surface, modulated by protonation states. Together, the pH‐dependent WGM and SERS results, supported by DFT calculations, establish a consistent mechanistic picture in which GLP binding strength, coordination geometry, and reversibility are dictated by protonation‐controlled functional group availability. This integrated experimental–theoretical approach enables direct validation of molecular binding energetics at nanostructured interfaces and highlights the utility of optoplasmonic sensing for resolving competitive, pH‐regulated adsorption processes at the single‐molecule level.

DFT calculations using an Au_22_(111) cluster model were used to compare pH‐dependent GLP–Au interaction energies and coordination configurations (Figure 2). The model is used to interpret relative trends rather than absolute adsorption free energies at the fully solvated CTAB‐coated AuNR interface. Full model construction and computational details are provided in the Supporting Information [32, 33]. The calculated interaction energies reveal a strong pH dependence, with GLP binding strength increasing markedly under alkaline conditions. At pH 10.6, GLP exhibits the strongest binding, with an interaction energy of −176.5 kcal mol^−^^1^, associated with cooperative coordination of multiple functional groups (PO_3_^2^^−^, COO^−^, and:NH). At pH 9.2, binding strength is reduced to −89.2 kcal mol^−^^1^ and primarily involves coordination via PO_3_^2^^−^ and COO^−^, while at pH 5.6 the interaction weakens further to −38 kcal mol^−^^1^. At pH 2.6, GLP exhibits only minimal interaction with the Au surface.

### 2.2 Ca^2^^+^ Chelation Uncovers the Role of Buffer Molecules and Amine‐Driven Cooperative Coordination in GLP Binding

We next tested the DFT predicted role of GLP coordination by introducing Ca^2^^+^. The calculations indicate that Ca^2^^+^ complexation alters the GLP coordination environment and weakens the calculated GLP– Au_22_(111) interaction (Figure 2c), providing an experimentally testable perturbation of the proposed binding mechanism.

**FIGURE 3:**
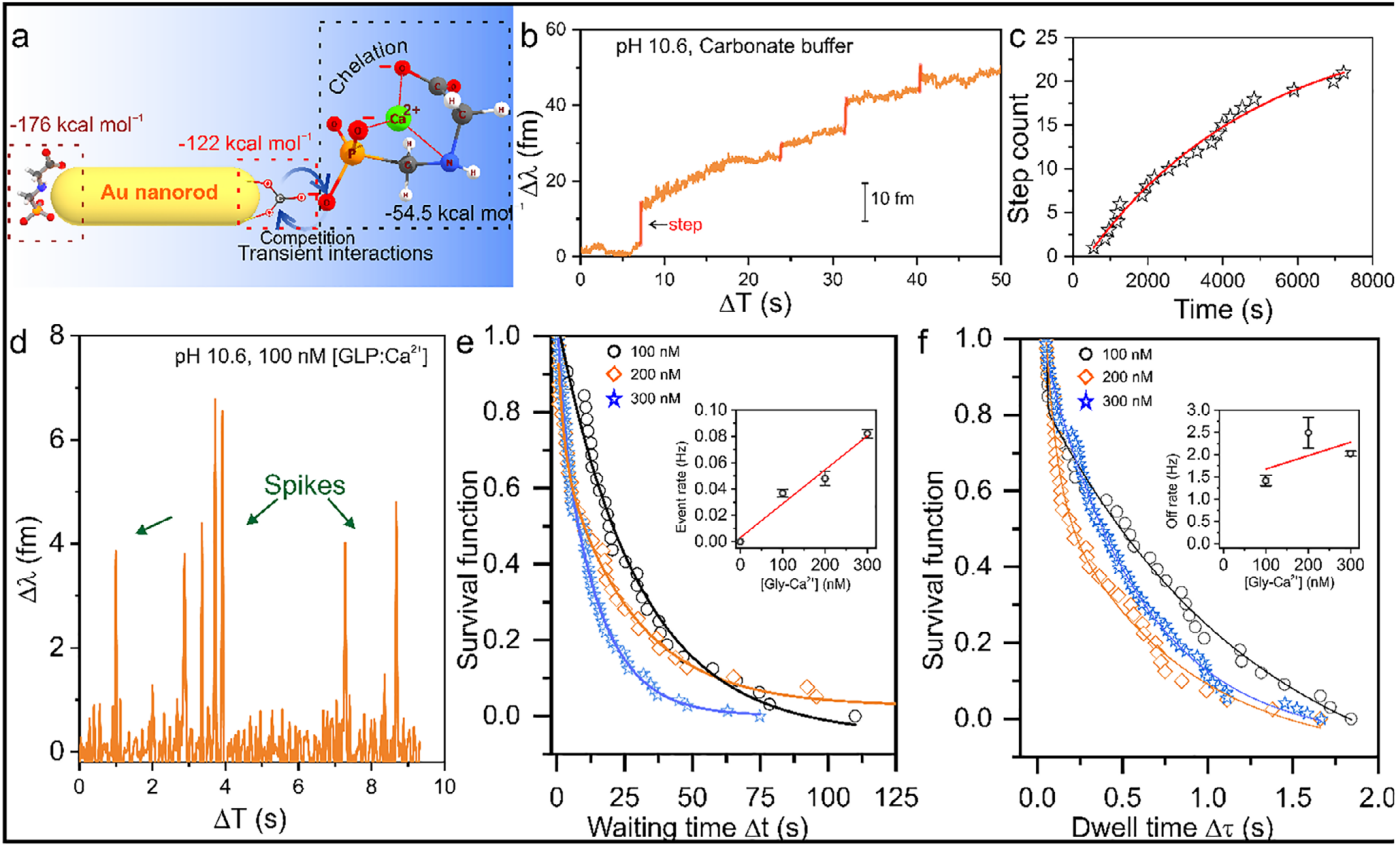
Modulation of GLP–Au interactions by Ca^2^^+^ chelation. (a) Schematic representation of GLP, [Ca^2^^+^–GLP], and carbonate interactions at the AuNR surface, with DFT‐calculated complexation energies (kcal mol^−^^1^). (b) Representative WGM resonance time trace (Δλ) for 100 nM GLP in carbonate buffer at pH 10.6. (c) Cumulative GLP binding‐event count as a function of time, with an exponential fit (red line). (d) Representative WGM resonance trace for 100 nM [Ca^2^^+^–GLP] at pH 10.6. (e) Survival functions of the waiting time (Δt) between consecutive [Ca^2^^+^–GLP] interaction events at 100, 200, and 300 nM, with exponential fits. Inset: event rate as a function of [Ca^2^^+^–GLP] concentration. (f) Survival functions of the event dwell time (Δτ) at 100, 200, and 300 nM [Ca^2^^+^–GLP], with exponential fits. Inset: off‐rate as a function of [Ca^2^^+^–GLP] concentration. Experiments were conducted at room temperature.

To test this hypothesis, a 1:1 molar complex of Ca^2^^+^ and GLP was prepared at pH 10.6 and its interaction with AuNR was monitored. In contrast to the sustained step‐like signals observed for free GLP (Figure 3b), the [Ca^2^^+^–GLP] complex produced short‐lived, spike‐like resonance shifts (Figure 3d), indicative of transient interaction events. This marked transition from stable to transient interactions demonstrates that Ca^2^^+^ chelation significantly reduces GLP's binding affinity for the Au surface (Figure 2c). The −54.5 kcal mol^−^^1^ binding energy of the GLP–Ca^2^^+^ complex on Au (see Figure 2c) is substantially lower than the −176 kcal mol^−^^1^ value for GLP–Au. The experiment was conducted in carbonate buffer, and carbonate species are known to bind to gold with a binding affinity of −122 kcal mol^−^^1^ (Figure 2). It is therefore proposed that the carbonate competes with GLP at the gold surface and that this competition between buffer molecules and the ligand gives rise to the transient, spike‐like signals observed for the GLP–Ca^2^^+^ complex at the Au surface (Figure 3d). The influence of buffer competition will be examined in the next section.

Statistical analysis of the transient binding events for Ca^2+^‐GLP at pH 10.6 in carbonate buffer follows a Poisson distribution for *K* spike events occurring within a time interval *T*, as expressed by:

$$p\left(K\right)=\frac{{\left(R\;\Delta T\right)}^{K}}{K!}\;e^{-R\;T}$$

where, *R* is the event rate. Figure 3e shows the survival function for the [Ca^2+^‐GLP] complex at concentrations of 100, 200, and 300 nM as a function of waiting time. The event rate increases linearly with [Ca^2^^+^–GLP] concentration (Figure 3e, inset; R^2^ = 0.926), indicating that the observed signals arise predominantly from the introduced complex and that the adsorption kinetics exhibit first‐order behavior over the investigated concentration range. In contrast, analysis of the survival function versus dwell time reveals that the dissociation rate constant (*k_off_*) is independent of analyte concentration under these conditions (Figure 3f, inset).

### 2.3 DFT and WGM Investigation of Competitive Adsorption Between Buffers and GLP

**FIGURE 4:**
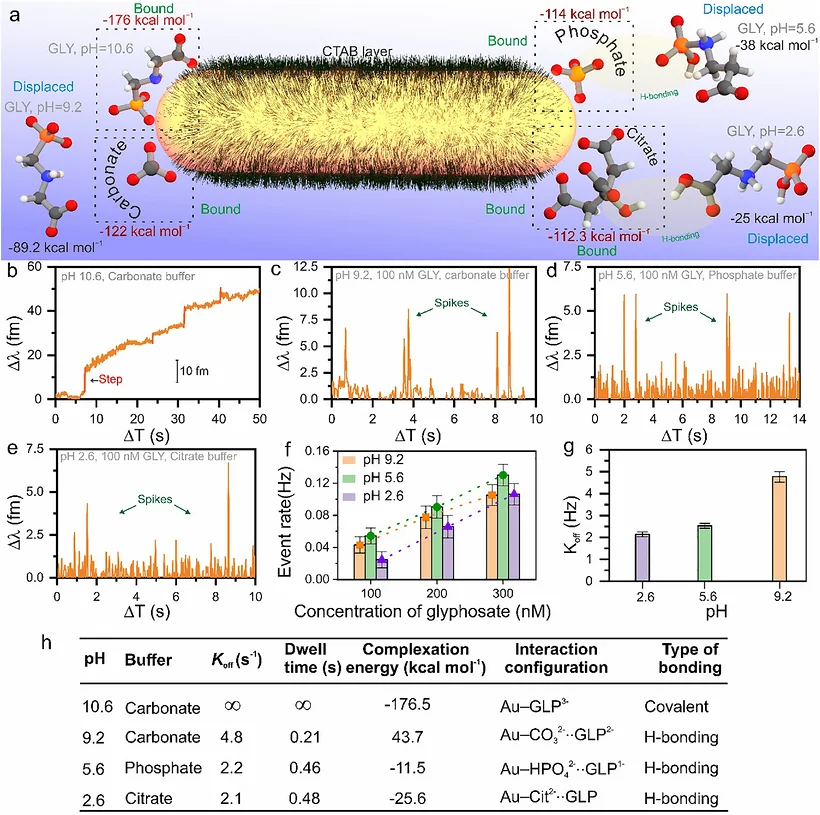
Competitive adsorption and pH‐dependent GLP–AuNR interactions in different buffer environments. (a) Schematic representation of competitive adsorption between GLP and buffer anions at the AuNR surface, with DFT‐calculated complexation energies (kcal mol^−^^1^). (b–e) Representative WGM resonance time traces of GLP–AuNR interactions at pH 10.6 in carbonate buffer (b), pH 9.2 in carbonate buffer (c), pH 5.6 in phosphate buffer (d), and pH 2.6 in citrate buffer (e). (f) Event rate as a function of GLP concentration (100, 200, and 300 nM) at pH 9.2, 5.6, and 2.6. (g) Extracted dissociation rate constants (koff) at pH 2.6, 5.6, and 9.2. (h) Summary of the kinetic parameters, DFT‐calculated complexation energies, interaction configurations, and proposed binding modes under the investigated pH and buffer conditions. Experiments were conducted at room temperature.

To elucidate the reaction mechanism and the role of pH and buffer molecules in regulating GLP–Au interactions, the solution pH was systematically adjusted using carbonate (pH 9.2), phosphate (pH 5.6), and citrate (pH 2.6) buffers. These buffers were deliberately chosen for their oxyanionic functionalities, which exhibit affinity for gold surfaces and pre‐occupy Au nanorod (AuNR) binding sites prior to GLP introduction. This strategy establishes competitive adsorption conditions that enable interrogation of reversible GLP physisorption dynamics. WGM resonance traces reveal predominantly transient, spike‐like signals at pH 9.2 (Figure 4c), with analogous behaviour observed at pH 5.6 and 2.6 (Figure 4d,e). These signatures indicate weak, reversible interactions and contrast sharply with the permanent binding observed under alkaline, buffer‐free conditions. The transient behavior arises from buffer‐induced passivation of Au binding sites: citrate, phosphate, and carbonate adsorb to the Au surface, imparting a net negative charge that generates electrostatic repulsion and suppresses strong GLP adsorption (Figure 4a). Event rates were quantified by varying GLP concentration (100–300 nM) across all pH regimes. Survival‐function analysis of the waiting time shows a linear increase in spike‐event rate with GLP concentration (Figure S10), supporting the assignment of the detected signals to GLP–Au interactions. In contrast, buffer adsorption events themselves remain undetected, likely due to their lower molecular polarizability, rapid diffusion and rapid passivation of the AuNR surface preclude resolvable WGM signals at the current sensitivity and time resolution (ms) (Figure S11).

To assess competitive adsorption, complexation energies of common buffer anions were calculated (Figure 2c). Carbonate (CO_3_^2−^) binds to Au_22_(111) surface with an energy of −122.1 kcal mol^−1^, while phosphate (HPO_4_^2^^−^) and citrate exhibit interaction energies of −114 and −112.3 kcal mol^−^^1^, respectively. At pH 10.6, GLP binds more strongly to the Au surface than carbonate, explaining the experimentally observed permanent binding events even in carbonate‐buffered solutions. The stronger binding energy of GLP relative to CO_3_^2−^ supports the conclusion that GLP displaces buffer molecules and form stable, permanent interactions with the Au surface. The persistence of strong GLP adsorption at pH 10.6 despite carbonate competition indicates that cooperative amine–oxyanion coordination outweighs buffer–Au interactions. This conclusion is further supported by the saturation behavior observed in the WGM experiments (Figure 3c), consistent with irreversible binding under alkaline conditions. In contrast, at pH 9.2, 5.6, and 2.6, adsorption of carbonate, phosphate, and citrate competitively passivates Au surface sites, reducing GLP affinity and resulting in transient binding dynamics. Control experiments using pure‐buffer solutions showed no discrete WGM binding events, confirming that the observed responses arise from GLP–Au interactions rather than nonspecific effects of the buffer environment (Figure S11). Consistent with these observations, DFT‐calculated interaction energies indicate that GLP cannot effectively displace buffer molecules at these pH values, favoring short‐lived encounters rather than stable adsorption. Collectively, these results demonstrate that pH‐dependent functional‐group availability, particularly participation of the amine group, governs GLP interaction strength and competitive displacement of buffer species at gold surfaces, providing a mechanistic framework that unifies theoretical predictions with single‐molecule experimental observations.

### 2.4 Dwell‐time Analysis of Transient GLP interactions in the Presence of Buffer Molecules

GLP binding affinity was further assessed by extracting off‐rate (*k_off_*) from survival function plot of interaction dwell times across all pH levels. The *k_off_* remained consistent within each pH regime, and were independent of concentration, suggesting intrinsic dissociation kinetics governed by GLP's ionic state. The dwell time measured at different pH values can therefore be used to analyse the binding affinity of the various ionic states of GLP toward the Au surface. At 100 nM GLP, *k*_off_ values were 4.8 s^−^^1^ (pH 9.2), 2.2 s^−^^1^ (pH 5.6), and 2.1 s^−^^1^ (pH 2.6) (Figure 4g), revealing a clear pH‐dependent trend. Overall, the magnitude of the dwell time correlates with the DFT‐calculated interaction (complexation) energy between GLP and the surface‐adsorbed buffer species (see Figure 4h and SI). Representative DFT‐optimised structures (Figure S13) illustrate GLP forming weak, non‐covalent contacts with the buffer molecules, consistent with the short‐lived, spike‐like resonance shifts observed in the WGM experiments.

Specifically, longer dwell times at pH 2.6 are attributed to reduced electrostatic repulsion and favorable hydrogen bonding interactions involving protonated phosphonate species (RPO^−^ oxyanion) and GLP, whereas increased negative charge at pH 9.2 enhances repulsion from the buffer‐passivated Au surface, leading to faster dissociation. This behavior correlates with the Au surface's isoelectric point and GLP's net charge. At pH 2.6 and 5.6, the Au surface is positively charged [34], while buffer molecules impart a net negative charge to the nanorods. GLP's net charge shifts from zwitterionic (0) at pH 2.6 to −2 at pH 9.2. At higher pH, increased negative charge enhances electrostatic repulsion with the passivated Au surface, elevating *k*_off_. Conversely, at pH 2.6, the reduced repulsion facilitates longer dwell times and stronger interactions. These findings highlight the key role of buffer‐induced passivation in modulating GLP‐Au binding affinity across pH regimes. The interplay between GLP's ionic state, buffer competition, and surface charge offers critical insights into the dynamics of analyte‐nanoparticle interactions, with implications for sensor design and nanoscale interfacial chemistry.

These findings suggest that dwell‐time analysis of single‐molecule interactions can provide quantitative insight into transient adsorption processes. As a supplementary analysis, fitting the dwell‐time distributions to complexation energies using an Arrhenius model (Figure S14) reveals that the interaction kinetics follow an expected binding energy dependence, reinforcing the notion that the off‐rates reflect energy barriers associated with buffer‐mediated physisorption rather than intrinsic molecular desorption from the gold surface.

## 3 Conclusion

In conclusion, this study demonstrates an optoplasmonic platform for label‐free, real‐time investigation of nanoparticle–molecule interactions at the single‐molecule level. By integrating single‐molecule WGM sensing with DFT calculations and complementary SERS measurements, we elucidate the pH‐dependent binding behavior of GLP at Au nanointerfaces. The results demonstrate that the protonation state of GLP strongly influences its interaction with Au, while buffer composition provides an additional competitive adsorption pathway that modulates the observed binding dynamics. DFT calculations indicate that GLP interacts more strongly with the Au surface at alkaline pH, with the strongest calculated interaction energy of −176.5 kcal mol^−^^1^ at pH 10.6, enabling GLP to compete effectively with carbonate for accessible Au binding sites. Ca^2^^+^ chelation weakens the GLP–Au interaction and is accompanied by a transition from persistent step‐like to transient spike‐like WGM events. At lower pH, competitive adsorption of carbonate, phosphate, and citrate is associated with predominantly transient GLP interactions.

The experimentally determined dwell times and dissociation rate constants (k_off_) show systematic trends with the DFT‐calculated interaction energies, while complementary SERS measurements support the proposed pH‐dependent changes in GLP coordination. Together, these observations show that buffer composition should not be regarded solely as an inert means of controlling pH in surface‐based single‐molecule measurements. Buffer species can interact with the sensing interface, compete with the target analyte for accessible binding sites, and consequently influence measured event rates, dwell times, and binding behavior. Buffer selection should therefore consider not only buffering capacity and target pH, but also buffer–surface affinity, analyte protonation state, ionic environment, and potential competition with the molecular recognition pathway. Where experimentally feasible, buffer‐free or weakly interacting electrolyte controls can help distinguish intrinsic analyte–surface interactions from buffer‐mediated effects.

More broadly, combining single‐molecule kinetic measurements with theoretical calculations of competing surface interactions and complementary spectroscopic characterization provides a useful approach for interrogating molecular interactions at nanointerfaces. The present findings provide practical guidance for the design and interpretation of future single‐molecule sensing experiments, particularly with respect to buffer selection and interfacial competition. However, because the present study focuses on GLP and CTAB‐stabilized AuNRs, the extent to which these considerations apply to other analytes, surface ligands, buffer chemistries, and nanomaterial platforms remains to be established experimentally.

## Author Contributions

**Frank Vollmer**: conceptualization, methodology, supervision, writing – review and editing, funding acquisition. **Srikanth Pedireddy**: conceptualization, methodology, investigation, validation, visualization, formal analysis, supervision, project administration, writing – review and editing, writing – original draft. **Kulathunga Mudalige Kalani Perera**: investigation, formal analysis, visualization, methodology, writing – original draft. **Sivaraman Subramanian**: methodology, visualization.

## Conflicts of Interest

The authors declare no conflicts of interest.

## Supporting information




**Supporting File**: advs77567‐sup‐0001‐SuppMat.docx.

## Data Availability

The data that support the findings of this study are available from the corresponding author upon reasonable request.

## References

[1] M. Falahati , F. Attar , M. Sharifi , et al., “Gold Nanomaterials as Key Suppliers in Biological and Chemical Sensing, Catalysis, and Medicine,” Biochimica et Biophysica Acta (BBA)—General Subjects 1864, no. 1 (2020): 129435, 10.1016/j.bbagen.2019.129435. 31526869

[2] V. Ramalingam , “Multifunctionality of Gold Nanoparticles: Plausible and Convincing Properties,” Advances in Colloid and Interface Science 271 (2019): 101989, 10.1016/j.cis.2019.101989. 31330396

[3] K. Siriwardana , A. LaCour , and D. Zhang , “Critical Sequence Dependence in Multicomponent Ligand Binding to Gold Nanoparticles,” The Journal of Physical Chemistry C 120, no. 12 (2016): 6900–6905, 10.1021/acs.jpcc.6b01202.

[4] T. Zhou , C. Hu , K. He , and Z. Li , “Expanding the Toolbox of Oxidants: Controllable Etching of Ultrasmall Au Nanoparticles Toward Tailorable NIR‐II Luminescence and Ligand‐Mediated Biodistribution and Clearance,” Analytical Chemistry 96, no. 44 (2024): 17840–17849, 10.1021/acs.analchem.4c04326. 39432839

[5] K. M. Greskovich , K. M. Powderly , M. M. Kincanon , et al., “The Landscape of Gold Nanocrystal Surface Chemistry,” Accounts of Chemical Research 56, no. 12 (2023): 1553–1564, 10.1021/acs.accounts.3c00109. 37260281

[6] S. Vincent , S. Subramanian , and F. Vollmer , “Optoplasmonic Characterisation of Reversible Disulfide Interactions at Single Thiol Sites in the Attomolar Regime,” Nature Communications 11, no. 1 (2020): 2043, 10.1038/s41467-020-15822-8. PMC7184569 32341342

[7] J. A. Olmos‐Asar , M. Ludueña , and M. M. Mariscal , “Monolayer Protected Gold Nanoparticles: The Effect of the Headgroup–Au Interaction,” Physical Chemistry Chemical Physics 16, no. 30 (2014): 15979–15987, 10.1039/C4CP01963F. 24963610

[8] M. Ben Haddada , J. Blanchard , S. Casale , et al., “Optimizing the Immobilization of Gold Nanoparticles on Functionalized Silicon Surfaces: Amine‐ vs Thiol‐Terminated Silane,” Gold Bulletin 46, no. 4 (2013): 335–341, 10.1007/s13404-013-0120-y.

[9] J. Zheng , Z. Zhu , H. Chen , and Z. Liu , “Nanopatterned Assembling of Colloidal Gold Nanoparticles on Silicon,” Langmuir 16, no. 10 (2000): 4409–4412, 10.1021/la991332o.

[10] D. Aureau , Y. Varin , K. Roodenko , O. Seitz , O. Pluchery , and Y. J. Chabal , “Controlled Deposition of Gold Nanoparticles on Well‐Defined Organic Monolayer Grafted on Silicon Surfaces,” The Journal of Physical Chemistry C 114, no. 33 (2010): 14180–14186, 10.1021/jp104183m.

[11] S. K. Ghosh , S. Nath , S. Kundu , K. Esumi , and T. Pal , “Solvent and Ligand Effects on the Localized Surface Plasmon Resonance (LSPR) of Gold Colloids,” The Journal of Physical Chemistry B 108, no. 37 (2004): 13963–13971, 10.1021/jp047021q.

[12] A. Maity , D. Bagchi , H. Tabassum , P. Nath , S. Sinha , and A. Chakraborty , “Diverse Role of Buffer Mediums and Protein Concentrations to Mediate the Multimodal Interaction of Phenylalanine‐Functionalized Gold Nanoparticle and Lysozyme Protein at Same Nominal pH,” The Journal of Physical Chemistry B 128, no. 43 (2024): 10625–10635, 10.1021/acs.jpcb.4c05463. 39440610

[13] N. Leo , J. Liu , I. Archbold , Y. Tang , and X. Zeng , “Ionic Strength, Surface Charge, and Packing Density Effects on the Properties of Peptide Self‐Assembled Monolayers,” Langmuir 33, no. 8 (2017): 2050–2058, 10.1021/acs.langmuir.6b04038. 28135097

[14] E. Zossimova , C. Jones , K. M. K. Perera , S. Pedireddy , M. Walter , and F. Vollmer , “Whispering gallery mode sensing Through the lens of quantum optics, artificial intelligence, and nanoscale catalysis,” Applied Physics Letters 125, no. 3 (2024): 1–13, 10.1063/5.0216468.

[15] Y. Lyu , L. M. Becerril , M. Vanzan , et al., “The Interaction of Amines With Gold Nanoparticles,” Advanced Materials 36, no. 10 (2024): 2211624, 10.1002/adma.202211624. 36952309

[16] X. Zhu , Q. Yang , J. Huang , I. Suzuki , and G. Li , “Colorimetric Study of the Interaction Between Gold Nanoparticles and a Series of Amino Acids,” Journal of Nanoscience and Nanotechnology 8, no. 1 (2008): 353–357, 10.1166/jnn.2008.18139. 18468082

[17] A. S. Dileseigres , Y. Prado , and O. Pluchery , “How to Use Localized Surface Plasmon for Monitoring the Adsorption of Thiol Molecules on Gold Nanoparticles?,” Nanomaterials 12 (2022): 1–15, 10.3390/nano12020292. PMC8778005 35055309

[18] K.‐H. Lee , S.‐J. Chen , J.‐Y. Jeng , Y.‐C. Cheng , J.‐T. Shiea , and H.‐T. Chang , “Fluorescence and Interactions with Thiol Compounds of Nile Red‐Adsorbed Gold Nanoparticles,” Journal of Colloid and Interface Science 307, no. 2 (2007): 340–348, 10.1016/j.jcis.2006.12.013. 17207807

[19] R. Chadha , A. Das , S. Kapoor , and N. Maiti , “Surface‐Induced Dimerization of 2‐Thiazoline‐2‐Thiol on Silver and Gold Nanoparticles: A Surface Enhanced Raman Scattering (SERS) and Density Functional Theoretical (DFT) Study,” Journal of Molecular Liquids 322 (2021): 114536, 10.1016/j.molliq.2020.114536.

[20] F. Madzharova , Z. Heiner , and J. Kneipp , “Surface‐Enhanced Hyper Raman Spectra of Aromatic Thiols on Gold and Silver Nanoparticles,” The Journal of Physical Chemistry C 124, no. 11 (2020): 6233–6241, 10.1021/acs.jpcc.0c00294. PMC7208179 32395194

[21] X. Zhang , Y. Yin , J. Wang , et al., “Switchable Photon‐Plasmon Coupling in a Single‐Nanoparticle‐Perturbed Microcavity,” ACS Photonics 12, no. 7 (2025): 3880–3885, 10.1021/acsphotonics.5c00943.

[22] Y. Chen , Y. Yin , L. Ma , and O. G. Schmidt , “Recent Progress on Optoplasmonic Whispering‐Gallery‐Mode Microcavities,” Advanced Optical Materials 9, no. 12 (2021): 2100143, 10.1002/adom.202100143.

[23] F. Vollmer and S. Arnold , “Whispering‐Gallery‐Mode Biosensing: Label‐Free Detection Down to Single Molecules,” Nature Methods 5, no. 7 (2008): 591–596, 10.1038/nmeth.1221. 18587317

[24] M. C. Houghton , S. V. Kashanian , T. L. Derrien , K. Masuda , and F. Vollmer , “Whispering‐Gallery Mode Optoplasmonic Microcavities: From Advanced Single‐Molecule Sensors and Microlasers to Applications in Synthetic Biology,” ACS Photonics 11, no. 3 (2024): 892–903, 10.1021/acsphotonics.3c01570. 38523742 PMC10958601

[25] P. Sprankle , W. F. Meggitt , and D. Penner , “Adsorption, Mobility, and Microbial Degradation of Glyphosate in the Soil,” Weed Science 23, no. 3 (1975): 229–234, 10.1017/S0043174500052929.

[26] M. M. Peixoto , G. F. Bauerfeldt , M. H. Herbst , M. S. Pereira , and C. O. da Silva , “Study of the Stepwise Deprotonation Reactions of Glyphosate and the Corresponding pKa Values in Aqueous Solution,” The Journal of Physical Chemistry A 119, no. 21 (2015): 5241–5249, 10.1021/jp5099552. 25629880

[27] E. Galicia‐Andrés , D. Tunega , M. H. Gerzabek , and C. Oostenbrink , “On Glyphosate–Kaolinite Surface Interactions. A Molecular Dynamic Study,” European Journal of Soil Science 72, no. 3 (2021): 1231–1242, 10.1111/ejss.12971. 34220276 PMC8247318

[28] M. Rigobello‐Masini , E. A. O. Pereira , G. Abate , and J. C. Masini , “Solid‐Phase Extraction of Glyphosate in the Analyses of Environmental, Plant, and Food Samples,” Chromatographia 82, no. 8 (2019): 1121–1138, 10.1007/s10337-019-03748-3.

[29] E. Zossimova , J. Fiedler , F. Vollmer , and M. Walter , “Hybrid Quantum‐Classical Polarizability Model for Single Molecule Biosensing,” Nanoscale 16, no. 11 (2024): 5820–5828, 10.1039/D3NR05396B. 38436120

[30] S. Arnold , M. Khoshsima , I. Teraoka , S. Holler , and F. Vollmer , “Shift of Whispering‐Gallery Modes in Microspheres by Protein Adsorption,” Optics Letters 28, no. 4 (2003): 272–274, 10.1364/OL.28.000272. 12653369

[31] F. Vollmer , D. Braun , A. Libchaber , M. Khoshsima , I. Teraoka , and S. Arnold , “Protein Detection by Optical Shift of a Resonant Microcavity,” Applied Physics Letters 80, no. 21 (2002): 4057–4059, 10.1063/1.1482797.

[32] J. R. B. Gomes and F. Illas , “The Adsorption of Methyl Nitrite on the Au(111) Surface,” Catalysis Letters 71, no. 1‐2 (2001): 31–35, 10.1023/A:1016644006002.

[33] T. Jadoon , K. Carter‐Fenk , M. B. A. Siddique , et al., “Silver Clusters Tune up Electronic Properties of Graphene Nanoflakes: A Comprehensive Theoretical Study,” Journal of Molecular Liquids 297 (2020): 111902, 10.1016/j.molliq.2019.111902.

[34] A. Carone , S. Emilsson , P. Mariani , A. Désert , and S. Parola , “Gold Nanoparticle Shape Dependence of Colloidal Stability Domains,” Nanoscale Advances 5 (2023): 2017–2026, 10.1039/D2NA00809B. 36998666 PMC10044300

