# Supplementary material for: WGM Single‐Molecule Validation of pH‐Dependent Molecular Energetics at Gold Nanointerfaces: Influence of Buffer Molecules on Glyphosate Ligand Binding
Source: Adv Sci (Weinh). 2026 Sep 14:e77567. Online ahead of print. doi: 10.1002/advs.77567 (PMC13574108; doi:10.1002/advs.77567)
Supplement: Supplementary file 1 [file ADVS-9999-e77567-s001.docx]

*Supporting information for*

**WGM Single-Molecule Validation of pH-Dependent Molecular Energetics at Gold Nanointerfaces: Influence of Buffer Molecules on Glyphosate Ligand Binding**

Kulathunga Mudalige Kalani Perera^1†^, Srikanth Pedireddy^1†*^, Sivaraman Subramanian^1^, Frank Vollmer^1*^

^1^Department of Physics and Astronomy, Living Systems Institute, University of Exeter, Exeter, EX4 4QD, UK

^*^[f.vollmer@exeter.ac.uk](mailto:f.vollmer@exeter.ac.uk), s.pedireddy@exeter.ac.uk

^†^ These authors contributed equally to this work

**WGM Setup**

WGM excitation was performed using an external cavity diode laser (Toptica TA Pro 642 HP, Toptica GmbH, Munich, Germany) at a nominal wavelength of λ = 642 nm, coupled to the microsphere via total internal reflection at an N-SF11 prism surface. The glass WGM microcavity was fabricated by melting a commercial single-mode optical fibre using a pulsed CO₂ laser (10.6 μm emission line), with one end held under tension by a small ceramic ferrule. A tapered stem (width 10 μm, length 500 μm) was formed, followed by high-power irradiation to produce a spherical resonator (diameter 88–95 μm) at the taper end. Fine curvature adjustment was achieved using low-power laser pulses. Analyte solutions were loaded into a polydimethylsiloxane (PDMS) V-shaped sample chamber with a maximum holding volume of 400 μL.

**Preparation of WGM sensor**

A schematic of the WGM sensor setup is shown in Figure 1. The WGM microcavity is a spherical glass microsphere resonator (diameter 85–95 μm) formed at the tip of a single-mode optical fibre. CTAB-AuNRs (1 OD, 0.1 μL) were attached to the microsphere surface using a pH 1.7 HCl solution, following a previously reported protocol. AuNR attachment was monitored in real time, with 7–8 NRs immobilised per microsphere. After immobilisation, the residual solution was removed, and the chamber was rinsed three times with Milli-Q water to remove non-specifically adsorbed or freely diffusing nanoparticles. A background measurement was then performed to verify the absence of any spurious signals before analyte experiments.

**Computational details for DFT**

The Au surface was modelled using a 22-atom Au(111) lattice, Au_22_(111), generated by cleaving (111) facet from an Au crystal and optimising the geometry to a minimal energy state in vacuum. All geometric optimizations, frequency calculations, counterpoise (CP) corrections,^1^ basis set superposition error (BSSE) corrections and complexation energy computations were performed by using the Gaussian 16 series of programs.^2^ All structures were optimized in the gas phase at the (U)M06-2X level of theory^3^ in which all metal atoms were described with the lanl2dz basis set^4^ and 6-31g** basis used for all non-metallic atoms. Lanl2dz basis set treats explicitly the outer 5s^2^5p^6^5d^10^6s^1^ electrons in gold atoms by a double-zeta basis set while the inner core electrons were replaced by the relativistic effective core potential, RECP, of Hay and Wadt.^4^ For the geometry optimization and the corresponding frequency calculations, a tight convergence criterion and an ultrafine integration grid (99,590) were adopted. Au_22_(111) model may influence the absolute interaction energies but are not expected to alter the relative pH-dependent energetic trends obtained using the same computational framework.

**Facet and hotspot considerations**

The Au_22_(111) cluster employed here provides a representative model for evaluating relative pH-dependent changes in GLP–Au adsorption energetics rather than a complete facet-resolved representation of the AuNR. Experimentally, adsorption and detection are expected to be strongly biased toward the AuNR tip regions. The longitudinal surfaces are densely passivated by CTAB, restricting direct access of GLP to the underlying Au, whereas the tip regions are comparatively accessible. In addition, the longitudinal plasmon mode generates strongly localized electromagnetic hotspots at the AuNR tips (Figure S8), making the WGM response substantially more sensitive to molecules interacting in these regions. Consequently, even if occasional adsorption occurs at incompletely passivated side sites, its contribution to the measured single-molecule signal is expected to be strongly suppressed. Heterogeneity in the measured step amplitudes can instead arise from different adsorption positions within the spatially non-uniform tip hotspot and from local variations in atomic coordination and adsorption geometry. Thus, the present Au_22_(111) calculations capture the relative pH-dependent adsorption trends relevant to the experimentally dominant tip interactions, while explicit facet, edge, CTAB, and solvent-resolved calculations would be required for quantitative determination of site-specific adsorption energies.

**Influence of buffer concentration**

The present experiments were performed with approximately 7 AuNRs per WGM microsphere in 350 µL of 30 mM buffer, corresponding to approximately 6×10^18^ buffer species in the measurement chamber. Buffer molecules are therefore present in overwhelming excess relative to the finite number of accessible Au adsorption sites. At this concentration, the AuNR interface is expected to experience a continuously replenished buffer reservoir, and the relevant competition is governed primarily by surface occupancy and displacement energetics rather than by the absolute number of buffer molecules in bulk solution. Accordingly, the effect of carbonate concentration is unlikely to scale linearly at high millimolar concentrations: once accessible Au sites are substantially occupied, further increases in carbonate concentration may produce only limited changes, whereas decreasing carbonate concentration sufficiently should reduce surface passivation and increase the probability of direct GLP–Au interactions. Importantly, at pH 10.6, GLP can produce stable binding despite the enormous excess of carbonate because its calculated Au interaction energy (−176.5 kcal mol⁻¹) exceeds that of carbonate (−122.1 kcal mol⁻¹), whereas at pH 9.2 GLP–Au interaction (−89.2 kcal mol⁻¹) is less favourable. Thus, the observed binding regime reflects competitive surface energetics and GLP protonation state rather than the bulk concentration ratio alone.

**Influence of ionic strength.**

Changes in pH and buffer composition necessarily modify the ionic environment and may influence GLP–Au interactions through electrostatic screening. However, the observed binding behaviour cannot be explained solely by ionic-strength effects. In buffer-free experiments, pH was adjusted using HCl/NaOH in the presence of a common 20 mM NaCl background electrolyte, and pH-dependent GLP–Au binding persisted in the absence of competing buffer anions. DFT calculations further predict pronounced protonation-dependent changes in GLP–Au interaction energy, reaching −176.5 kcal mol⁻¹ at pH 10.6 when cooperative amine–oxyanion coordination becomes available. When carbonate, phosphate, or citrate buffers are introduced, their substantial Au affinities provide an additional competitive adsorption pathway that accounts for the transition toward transient interactions. Ionic strength may therefore modulate electrostatic screening and binding kinetics, but the combined experimental and computational results indicate that protonation-state-dependent GLP coordination and competitive buffer adsorption are the dominant factors governing the observed binding regimes.

**Characterization**

A JEOL 2100 Transmission Electron Microscope was used to collect images of AuNRs. The clusters' hydrodynamic radius and zeta potential were measured with dynamic light scattering (DLS) on a Malvern Zetasizer Ultra.

SERS spectra were acquired using a Renishaw inVia confocal Raman microscope equipped with a 785 nm excitation laser and a 50× long-working-distance objective (NA = 0.45). The laser power, integration time, and number of accumulations were kept constant for all measurements to enable direct comparison between experimental conditions. The thermally evaporated Au film was selected because its predominantly Au(111)-like surface provides a suitable model for probing the molecular interactions occurring at the exposed Au nanorod tips investigated by WGM sensing. The SERS measurements were used to identify pH-dependent changes in glyphosate coordination and to provide complementary spectroscopic evidence supporting the DFT-predicted binding configurations. Raman spectra of the bare Au substrate, buffer solutions, glyphosate solutions, and glyphosate in the presence of the respective buffer systems were collected under identical experimental conditions.

**WGM Sensor measurements**

WGM spectra were acquired by scanning the tunable laser across a narrow bandwidth centred on the resonance frequency at a scanning frequency of 50 Hz, corresponding to a time resolution of 20 ms (Figure 1C). Any perturbation of the local optical environment, arising from either AuNR attachment to the microsphere surface or analyte–AuNR interaction — induces a shift in the resonance mode position (red- or blue-shift). Prior to analyte introduction, AuNRs were immobilised onto the microsphere to enhance the local electromagnetic field and improve the signal-to-noise ratio of the sensor platform. The attachment of AuNRs to the microsphere surface produces a characteristic red-shift in the WGM resonance, as illustrated by the transition from the initial spectrum (orange) to the NR-functionalised spectrum (blue) in Figure 1C. Here, κ₀ and κ₁ denote the full-width-at-half-maximum (FWHM) of the resonance before and after AuNR’s immobilisation, respectively, and Δλ represents the corresponding shift in resonance position. Real-time sensor signals were obtained by continuously tracking both the WGM resonance position and FWHM over time. The magnitude of the resonance shift is proportional to the overlap integral of the local electric field intensity with the polarisability volume of the bound analyte. Two distinct signal types are characteristic of plasmonic WGM sensing: transient spikes and discrete permanent steps. Transient signals (spikes) arise from reversible physical adsorption and desorption events, reflecting the formation of unstable, non-covalent interactions between the analyte and the nanorod surface ligands. In contrast, discrete step like shifts in the resonance position are indicative of irreversible, covalent bond formation between the analyte molecule and the AuNR surface. Transient spike events can be interpreted within a binding kinetics framework as follows:

R+L RL

Where the R, L, k_on_, and k_off_ represent the AuNR surface site, analyte molecule, onset rate (M ^−1^ s^−1^), and offset rate (s^−1^), respectively. Transient spike events thus provide access to quantitative binding kinetics, whereas discrete steps report exclusively on the surface chemistry between the analyte and AuNR. To ensure cross-experiment comparability, a constant number of AuNRs (7–8) was immobilised per microsphere, minimising variability in the number of available sensing sites. This platform thereby enables systematic investigation of analyte binding as a function of solution pH, analyte concentration, temperature, and electrolyte composition.

***Figure S1.*** (A) Schematic of the experimental setup for optical detection using a whispering-gallery-mode (WGM) optoplasmonic sensor and a tunable laser source (λ = 640 nm). The prism-based configuration monitors interactions within an aqueous solution housed in a PDMS chamber. The setup includes collimated light, a photodetector, and an analyte solution. (B) Representative transmission electron microscopy (TEM) image of the nanoparticle, revealing dimensions of 25 × 10 nm. (C) Spectral shifts in transmission (Tr) as a function of wavelength for two states, highlighting the spectral shift Δλ due to molecular interactions. K₀ and K₀ + δK correspond to the initial and shifted resonance peak positions. (D) Temporal monitoring of Δλ for interaction dynamics: (i) Stepwise changes in Δλ over time due to molecular events. (ii) Analysis of key parameters, including the time difference between consecutive events (ΔT), interaction duration on the nanosurface (Δꞇ), and spike heights (h). (E) Noise analysis of Δλ and Δκ over time, showing signal stability within 3σ limits, ensuring the reliability and precision of spectral shift measurements under experimental conditions.

Spike events were defined as signals exceeding a 3–5σ threshold above the background standard deviation (σ), where σ was estimated by segmenting the WGM time trace into windows of N points and computing the standard deviation of each window. The minimum observed σ was 0.4–0.5 fm. All spike events above the threshold were analysed manually, with each event characterised by its amplitude (h), dwell time (τ), and inter-event separation time (ΔT).

***Figure S2***. Morphological and structural characterization of the AuNRs. (a) Representative TEM, and (b) high-resolution TEM (HRTEM) images of the AuNRs, showing their uniform rod-like morphology and rounded ends. (c) Statistical analysis of the AuNR dimensions obtained from TEM images. The nanorods exhibit an average length of approximately 25 nm and an average width of approximately 10 nm.

***Figure S3.*** Optical characterization of AuNR attachment to the WGM microsphere. (a) Representative optical image of the silica WGM microsphere (diameter ≈86 µm) before AuNR immobilization. (b) Corresponding scattering image showing spatially localized scattering features on the microsphere after AuNR attachment. Selected scattering features are indicated by dashed circles. AuNR attachment was additionally monitored in real time through discrete step-like changes in the WGM resonance during the immobilization process.

**Calculation of molecular polarizabilities**

Molecular geometries were fully optimized in water without symmetry constraints at the PBE/aug-cc-pVTZ level of theory, employing the polarizable continuum model (PCM) to account for bulk solvent effects of water. Static molecular polarizabilities (α) were computed analytically for the optimized geometries using the Polar keyword at the same level of theory (PBE/aug-cc-pVTZ with PCM-water solvation).

**Estimation of WGM step heights from DFT-calculated molecular polarizabilities**

$$Shift in resonance wavelength (\Delta\lambda\text{)}=\frac{\alpha_{\text{ex}}\cdot\mid\mathbf{E}\mid^{2}/\mid\mathbf{E}_{0}\mid^{2}}{2n_{\text{med}}V_{\text{wgm}}}\cdot\lambda_{0}$$

$$=\frac{\alpha_{\text{ex}}}{{2V}_{\text{eff}}}\cdot\lambda_{0}$$

Refractive index of medium ($n_{\text{med}})=1.33$

Mode volume( $V_{\text{wgm}})$=mode volume=420 μm^3^

Effective mode volume ($V_{\text{eff}}$)=mode volume= 6.6*10^-4^ μm^3^

$\lambda_{0}=$642 nm

$\alpha_{\text{ex}}$= molecular polarizability(Å³) calculated using density functional theory (DFT)

***Table S1.*** Experimentally measured WGM step heights (averages over molecules landing randomly at the nanorod tip) compared with DFT-calculated molecular polarizabilities and predicted step heights at the tip of the nanorod. The estimated WGM step height column gives the maximum shift calculated from DFT polarizabilities (molecule at tip apex). Due to the cos²-shaped near-field intensity distribution along the nanorod tip, the expected average shift is half the maximum value (see text above), bringing the predicted and experimental values into good agreement.

| pH | Molecular polarizability (Å³): From DFT | Average WGM step height (fm): From experiment | Estimated maximum WGM step height (fm) **at apex of** nanorod tip: Using DFT values |
| --- | --- | --- | --- |
| 2.6 | 16.3 | 3±1 | 6 |
| 5.6 | 17.8 | 3.3±1 | 6.5 |
| 9.2 | 19.9 | 4±3 | 7.3 |
| 10.6 | 22.3 | 4.7±3 | 8.2 |

While the WGM sensor is intrinsically sensitive to changes in molecular polarisability upon binding,^5, 6^ the effective polarisability of GLP is not solely an intrinsic molecular property but is further modified by electrostatic interactions and charge redistribution arising from complexation with the Au surface. These interaction-induced contributions fall outside of what is usually considered in the conventional reactive sensing framework which relates WGM wavelength shift to excess polarisabilty of a molecule in water.^7, 8^ In this study, density functional theory is therefore employed to explicitly account for adsorption geometry, protonation state, and interfacial charge transfer.

Compared with measurements performed on proteins (enzymes) such as ADK and 3PGK,^9, 10^ which possess polarizabilities more than three orders of magnitude larger than small molecules such as GLP, our results indicate that the nanorod does not simply rescale the WGM mode volume by a fixed enhancement factor but instead seems to define distinct effective mode-volume regimes depending on analyte size. For small molecules (e.g. glyphosate, α ~22 Å³), the experimentally observed shifts are reconciled when the effective mode volume is identified with a volume like the plasmonic hotspot volume at the nanorod tip as demonstrated in this work.

| Mode volume | V_eff_ (μm^3^) | Estimated maximum WGM step height (fm) at apex of nanorod tip |
| --- | --- | --- |
| Evaluated at surface | 420 | 1.23x10^-5^ |
| Evaluated at surface with nanorod | 0.42 | 0.013 |
| Nanorod tip (10 nm) | 1×10^−6^ | 5392 |
| **Plasmonic hotspot**  **This paper** | **6.6×10^−4^** | **8.2** |

**Stability of CTAB-Coated AuNRs under the Investigated pH and Buffer Conditions**

To address the stability of the CTAB-coated AuNRs, we have additionally included pH-dependent zeta-potential measurements, UV–vis spectra, and visual stability observations under the experimentally relevant buffer conditions. The AuNRs retain a positive zeta potential across the investigated pH range, while preservation of the characteristic plasmon bands and the absence of visible precipitation or aggregation support their stability, including under alkaline conditions. Stable WGM resonances and reproducible binding responses further support the structural integrity of the immobilized AuNRs sensing platform throughout the experiments.

***Figure S4.*** (a) Visual assessment of CTAB-coated AuNR stability under different buffer and pH conditions. (b) UV–vis characterization of CTAB-coated AuNR stability under different buffer and pH conditions. (c) Zeta potential of CTAB-coated gold nanorods (AuNRs) under different buffer and pH conditions

**Figure S5.** a. Cumulative step count as a function of time for four experimental conditions: pH 2.6 with 20 mM NaCl (black squares), pH 5.6 with 20 mM NaCl (red circles), pH 9.2 with 50 mM NaCl (blue triangles), and pH 10.6 with 50 mM NaCl (green triangles). b Histogram of step heights with normal fits for each experimental condition.

***Figure S6.*** SERS control measurements on a thermally evaporated Au substrate.

***Figure S7***. DFT-simulated vibrational spectra of GLP on Au_22_(111) at different pH conditions, illustrating changes in spectral features arising from pH-dependent protonation states.

**Statistical Analysis**

Statistical analyses were performed on the WGM step-height distributions obtained at pH 2.6, 5.6, 9.2, and 10.6. Data are reported as mean ± standard deviation (SD), with the median and interquartile range (IQR) additionally used to describe the distributions. The numbers of individual binding events analysed were n = 49, 64, 35, and 54 for pH 2.6, 5.6, 9.2, and 10.6, respectively.

The normality of each step-height distribution was assessed using the Shapiro–Wilk test. As significant deviations from normality were observed for all four datasets (p < 0.05), differences among the four pH conditions were primarily evaluated using the Kruskal–Wallis test, a rank-based nonparametric test that does not assume normally distributed data. When the overall Kruskal–Wallis test was significant, pairwise comparisons were performed using two-sided Mann–Whitney U tests, with the Holm method applied to adjust p-values for multiple comparisons.

As a complementary parametric analysis, Welch's ANOVA and pairwise two-sided Welch's t-tests were also performed because these tests are more robust than conventional ANOVA and Student's t-tests when group variances and sample sizes are unequal. Holm correction was again applied to the pairwise comparisons. Statistical significance was defined as an adjusted p < 0.05. All statistical tests were two-sided.

For visualization, step-height distributions are presented as violin plots with embedded box plots, where the box represents the IQR (25^th^–75^th^ percentiles), the central marker represents the median, and the whiskers extend to values within 1.5 × IQR. Exact p-values are reported where appropriate.

**Estimation of average WGM shift from near-field intensity integration along the nanorod tip**

The WGM wavelength shift induced by a single molecule depends on the local near-field intensity |E|²/|E₀|² at the precise location where the molecule interacts with the nanorod tip. Since molecules land at random positions along the tip, the experimentally measured step heights represent an average over the near-field intensity distribution at the tip. To relate the DFT-calculated maximum WGM shift (corresponding to a molecule positioned at the point of peak field intensity at the nanorod tip) to the experimentally observed average shift, we evaluate the effective mean intensity by integrating the near-field intensity along the nanorod tip.

As shown in Figure S2, the near-field intensity distribution along the nanorod follows approximately a cosine profile, with the maximum at the tip apex. The effective mean intensity experienced by a randomly adsorbing molecule is therefore:

⟨|E|²/|E₀|²⟩ = ⟨cos²(θ)⟩ = 1/2

where θ is the angular coordinate along the nanorod tip. Since the average of cos² equals 1/2, the predicted average WGM wavelength shift is half the maximum calculated shift (i.e., the shift expected when a molecule binds precisely at the tip apex where the field is maximal). This factor of 1/2 therefore accounts for the approximately factor of 2 discrepancy that would otherwise be observed between the DFT-estimated maximum shift (from Table S1, Estimated WGM step height column) and the experimentally measured average step height. As shown in Table S1, once this averaging factor is applied, the average experimental WGM step heights agree well with the DFT predictions across all pH conditions investigated.

***Figure S8.*** Electric field distribution around AuNR and field distribution around the tip, obtained from COMSOL simulations.

***Figure S9.*** Statistical comparison of WGM step-height distributions at different pH values. Violin plots showing the distributions of WGM step heights measured at pH 2.6, 5.6, 9.2, and 10.6. The interquartile range (IQR; 25^th^–75^th^ percentile), median, and whiskers (1.5 × IQR) are indicated. Differences among the four pH conditions were assessed using the Kruskal–Wallis test (H(3) = 16.38, p = 9.48 × 10⁻⁴). Horizontal brackets indicate statistically significant pairwise comparisons, with the corresponding p-values shown.

**Spikes -pH 9.2 (Carbonate buffer), 5.6 (phosphate buffer) and 2.6 (citrate buffer) -statistical assessment**

***Figure S10.*** (a, d, g) Histograms of spike heights for glyphosate (GLP) at pH 9.2, 5.6, and 2.6, respectively, across different concentrations (100 nM, 200 nM, and 300 nM). The histograms include averaged shifted distributions, and the red solid line represents the spike detection threshold (3σ). (b, e, h) Survival functions of time separation (ΔT) between consecutive interaction events for GLP at varying concentrations (100 nM, 200 nM, and 300 nM) at pH 9.2, 5.6, and 2.6, respectively. Insets depict the on-rate (k_on_) as a function of GLP concentration, demonstrating linear behaviour with increasing concentration. (c, f, i) Survival functions of dwell time (Δτ) for GLP interactions with the gold surface under different pH conditions (pH 9.2, 5.6, and 2.6, respectively). Insets show the off-rate (k_off_) versus dwell time, which is independent of the GLP concentration, indicating stability in interaction dynamics.

Figure (a, d, and g) plots depict the statistical distribution of the spike height extracted from the trace data for pH 9.2, pH 5.2 and 2.6, respectively. Freedman Diaconi’s rule was selected as the bin method to build the Histogram plots of spike heights.

The Poisson distribution is a discrete probability distribution that shows the probability of the number of events (k) interested within a fixed time frame. The exponential distribution is closely related to the Poisson distribution for the number of observed events (in our scenario, binding or transient events), which describes the waiting time between two consecutive events obeying Poisson statistics. The statistical analysis was carried out, the time separation/ waiting time between interaction events to confirm the single molecule events in our system (figure 2 b, e, and h at pH 9.2,5.6 and 2.6, respectively). Furthermore, these plots show that it obeys the exponential statistics for single molecule spike events as expected.

Under this condition, the spikes (transient signals) follow the Poisson probability distribution where *k* number of spikes in the separation of time ∆T.

$$P(k,R,\Delta T)=\frac{{(R\Delta T)}^{k}}{k!}e^{-R\Delta T}$$

Where R means on rate (1/τ_c_) or event rate and extracted the R values for each of those cases, and the experiment was extended to study the concentration dependence of observed transient events, spikes. The determined average event rate increases linearly (R^2^ ~ 0.99) with the GLP, concentration, as shown in the inset of figure 2 Aii, Bii, and Cii at pH 9.2,5.6 and 2.6, respectively, as expected for single molecule events.

The plots of the survival function of the interaction dwell time τ (S(τ) = P(T > τ), where P(τ) is the probability function, and T is a continuous random variable in time for pH 9.2,5.6 and 2.6, respectively. An off-rate was extracted for these data, and the off-rate was independent of the GLP concentration (Figure 4). Moreover, the relatively lower off rate indicates the presence of electrostatic interaction or minimal repulsion between the GLP and the gold surface.

***Figure S11.*** WGM control measurements in buffer solutions without GLP. Representative wavelength-shift (Δλ) and differential (Δκ) traces obtained in citrate buffer at pH 2.6, phosphate buffer at pH 5.6, and carbonate buffer at pH 9.2 and 10.6 in the absence of GLP.

***Figure S12.*** pH-matched WGM control measurements in the absence of buffer and GLP. Representative wavelength-shift (Δλ) and differential (Δκ) traces obtained at pH 2.6, 5.6, 9.2, and 10.6 without buffer or GLP.

***Figure S13***. Illustration of GLP complexation mediated by buffer molecules with the Au(111) surface under varying pH conditions (a) 2.6, and (b) 5.6, as determined by density functional theory (DFT) calculations.

***Figure S14.*** Arrhenius analysis of dwell-time distributions. Dwell-time distributions obtained from experiments were fitted to DFT calculated complexation energies using an Arrhenius model.

**Coordinates**

Au_22_ cluster

79	2.063039000	2.419213000	-2.291919000

79	0.724545000	0.053344000	-2.867933000

79	2.016713000	4.372372000	-0.255470000

79	0.418378000	2.067715000	0.080210000

79	-0.890684000	-0.320893000	-0.482284000

79	3.608625000	0.101770000	-2.687471000

79	6.424671000	0.104365000	-2.133618000

79	5.127750000	-2.286958000	-2.635039000

79	4.910911000	2.419956000	-1.778808000

79	4.794631000	4.378907000	0.247091000

79	6.176033000	1.987334000	0.834516000

79	3.650395000	-4.612374000	-2.796819000

79	2.167285000	-2.301559000	-3.034633000

79	1.860304000	-0.450783000	0.315931000

79	4.640844000	-0.494648000	0.686991000

79	3.241065000	-2.911012000	0.309939000

79	3.234428000	1.913391000	0.896203000

79	1.782228000	-5.174218000	-0.762502000

79	0.468721000	-2.763044000	-0.677847000

79	7.509313000	-0.428264000	0.502096000

79	4.873467000	-5.227568000	-0.339148000

79	6.175038000	-2.836849000	0.033714000

Au_22_-GLP^-3^ (pH=10.6)

79	2.883791000	3.329902000	-2.354601000

79	1.441905000	0.832506000	-2.354606000

79	1.441898000	4.162320000	0.000011000

79	-0.000001000	1.664910000	-0.000012000

79	-1.441890000	-0.832497000	-0.000021000

79	4.325627000	0.832493000	-2.354616000

79	7.209334000	0.832499000	-2.354621000

79	5.767349000	-1.665095000	-2.354630000

79	5.767486000	3.329926000	-2.354607000

79	4.325597000	4.162330000	0.000017000

79	5.767469000	1.664955000	0.000028000

79	4.325646000	-4.162350000	-2.354601000

79	2.883799000	-1.664801000	-2.354629000

79	1.441920000	-0.832489000	-0.000009000

79	4.325699000	-0.832444000	0.000062000

79	2.883853000	-3.329897000	0.000000000

79	2.883789000	1.664904000	0.000023000

79	1.441906000	-5.827297000	-0.000011000

79	0.000019000	-3.329895000	-0.000026000

79	7.209371000	-0.832519000	0.000022000

79	4.325607000	-5.827327000	-0.000012000

79	5.767526000	-3.329935000	0.000036000

15	1.748162000	0.562095000	2.981836000

8	1.180784000	-0.683598000	2.229807000

8	1.737060000	1.806572000	2.010679000

8	1.154745000	0.835730000	4.334354000

8	3.856389000	-4.516483000	2.446405000

8	5.706917000	-3.238015000	2.561657000

7	4.134612000	-0.986314000	2.591396000

6	3.577493000	0.201471000	3.260315000

6	3.610144000	-2.243317000	3.136488000

6	4.465714000	-3.442214000	2.686182000

1	3.696531000	0.115070000	4.352856000

1	4.138379000	1.080502000	2.919654000

1	2.572268000	-2.368361000	2.823094000

1	3.620706000	-2.216308000	4.241982000

1	5.145309000	-1.021400000	2.730901000

Au_22_-GLP^-2^ (pH=9.2)

79	2.883798000	3.329889000	-2.354601000

79	1.441826000	0.832508000	-2.354589000

79	1.441900000	4.162303000	0.000007000

79	-0.000018000	1.664913000	-0.000003000

79	-1.441882000	-0.832468000	-0.000024000

79	4.325582000	0.832486000	-2.354599000

79	7.209393000	0.832501000	-2.354597000

79	5.767497000	-1.664904000	-2.354599000

79	5.767493000	3.329893000	-2.354601000

79	4.325602000	4.162297000	0.000002000

79	5.767495000	1.664894000	0.000012000

79	4.325614000	-4.162307000	-2.354592000

79	2.883783000	-1.664960000	-2.354582000

79	1.441954000	-0.832444000	-0.000003000

79	4.325606000	-0.832523000	0.000009000

79	2.883816000	-3.329901000	-0.000002000

79	2.883793000	1.664906000	0.000009000

79	1.441905000	-5.827295000	-0.000018000

79	0.000020000	-3.329877000	-0.000028000

79	7.209402000	-0.832506000	-0.000003000

79	4.325611000	-5.827302000	0.000002000

79	5.767511000	-3.329904000	0.000000000

15	2.075336000	0.718737000	3.093687000

8	2.964219000	1.746241000	2.337618000

8	0.857425000	1.219752000	3.802806000

8	1.956894000	-0.609813000	2.280498000

8	7.301428000	-2.432167000	2.946435000

8	6.910290000	-0.299769000	2.303263000

7	4.448158000	-0.427207000	3.426014000

6	3.367333000	0.096766000	4.318472000

6	5.562674000	-1.199115000	4.024218000

6	6.715443000	-1.353275000	2.995540000

1	2.998163000	-0.733654000	4.923260000

1	3.791270000	0.880806000	4.948361000

1	4.832113000	0.364148000	2.881537000

1	5.188254000	-2.175613000	4.331646000

1	5.916532000	-0.646040000	4.898597000

1	3.930334000	-0.993339000	2.725102000

Au_22_-GLP^-1^ (pH=5.2)

79	2.883800000	3.329901000	-2.354602000

79	1.441901000	0.832500000	-2.354601000

79	1.441898000	4.162299000	0.000000000

79	0.000000000	1.664899000	0.000003000

79	-1.441899000	-0.832501000	0.000001000

79	4.325602000	0.832501000	-2.354602000

79	7.209399000	0.832506000	-2.354598000

79	5.767521000	-1.664895000	-2.354599000

79	5.767501000	3.329903000	-2.354601000

79	4.325598000	4.162301000	-0.000001000

79	5.767500000	1.664903000	0.000003000

79	4.325604000	-4.162310000	-2.354602000

79	2.883806000	-1.664902000	-2.354603000

79	1.441901000	-0.832501000	0.000003000

79	4.325603000	-0.832498000	0.000002000

79	2.883796000	-3.329900000	0.000003000

79	2.883801000	1.664901000	0.000002000

79	1.441899000	-5.827300000	0.000008000

79	-0.000001000	-3.329901000	0.000003000

79	7.209400000	-0.832500000	-0.000008000

79	4.325600000	-5.827307000	-0.000005000

79	5.767471000	-3.329902000	-0.000004000

15	2.441234000	-0.094754000	3.652735000

8	3.990600000	0.104690000	3.111696000

8	2.033437000	0.943140000	4.628755000

8	1.572165000	-0.521621000	2.482329000

8	4.360128000	-5.897584000	2.485654000

8	5.415004000	-3.895411000	2.536301000

7	3.299879000	-2.623324000	3.394321000

6	2.818474000	-1.714493000	4.473459000

6	3.342814000	-4.088286000	3.658853000

6	4.495276000	-4.699458000	2.814237000

1	1.898236000	-2.128754000	4.889154000

1	3.586573000	-1.645856000	5.244615000

1	4.260753000	-2.363626000	3.101134000

1	2.384482000	-4.534876000	3.389548000

1	3.537245000	-4.240085000	4.723246000

1	4.090210000	0.871422000	2.526365000

1	2.705963000	-2.424675000	2.568706000

Au_22_-GLP (pH=2.6)

79	3.923261000	0.953283000	-1.345006000

79	1.223836000	1.758220000	-1.962651000

79	4.080331000	3.019269000	0.660790000

79	1.380950000	3.824178000	0.043120000

79	-1.318443000	4.629105000	-0.574493000

79	1.786281000	-0.981260000	-1.258208000

79	2.348707000	-3.720334000	-0.553764000

79	-0.350724000	-2.915440000	-1.171392000

79	4.485694000	-1.785917000	-0.640559000

79	4.642716000	0.280055000	1.365187000

79	2.505717000	-1.654315000	1.451993000

79	-3.050029000	-2.110531000	-1.789059000

79	-0.912671000	-0.175881000	-1.875852000

79	-0.756081000	1.889831000	0.129968000

79	-0.193979000	-0.849487000	0.834376000

79	-2.893035000	-0.044553000	0.216761000

79	1.943305000	1.084862000	0.747577000

79	-5.592425000	0.760354000	-0.400910000

79	-3.455440000	2.694741000	-0.487668000

79	0.368766000	-3.588682000	1.538831000

79	-5.030052000	-1.978850000	0.303526000

79	-2.330660000	-2.783829000	0.921166000

15	-0.538326000	0.723105000	4.026053000

8	-0.503919000	1.982328000	2.916532000

8	0.185967000	-0.463134000	3.462722000

8	-0.327578000	1.332792000	5.364325000

8	-6.433765000	2.606870000	2.494234000

8	-4.469753000	3.710604000	2.567924000

7	-3.086623000	1.573720000	3.367206000

6	-2.360350000	0.350893000	3.832725000

6	-4.553554000	1.420892000	3.256753000

6	-5.132378000	2.717684000	2.732930000

1	-2.770094000	0.060092000	4.802116000

1	-2.542798000	-0.434214000	3.095144000

1	-2.698485000	1.841402000	2.445031000

1	-4.969078000	1.174072000	4.236772000

1	-4.770309000	0.605538000	2.558228000

1	0.022785000	2.704594000	3.288957000

1	-6.745570000	3.444136000	2.113548000

1	-2.850992000	2.368457000	3.973483000

Au_22_-CO_3_^2-^

79	2.883818000	3.329899000	-2.354598000

79	1.442109000	0.832413000	-2.354696000

79	1.441894000	4.162218000	-0.000041000

79	0.000002000	1.664731000	-0.000028000

79	-1.442003000	-0.832618000	0.000104000

79	4.325711000	0.832549000	-2.354630000

79	7.209450000	0.832463000	-2.354642000

79	5.767535000	-1.664872000	-2.354604000

79	5.767536000	3.329916000	-2.354619000

79	4.325606000	4.162279000	-0.000012000

79	5.767556000	1.664908000	-0.000088000

79	4.325522000	-4.162189000	-2.354646000

79	2.883939000	-1.664240000	-2.354767000

79	1.441706000	-0.832958000	0.000122000

79	4.325618000	-0.832382000	-0.000003000

79	2.883771000	-3.329831000	0.000053000

79	2.883886000	1.664778000	-0.000039000

79	1.441861000	-5.827270000	0.000079000

79	-0.000222000	-3.329966000	0.000162000

79	7.209418000	-0.832476000	0.000046000

79	4.325526000	-5.827269000	-0.000005000

79	5.767461000	-3.329883000	0.000051000

8	1.207652000	0.140615000	2.036144000

8	3.417945000	0.652528000	1.939828000

6	2.343578000	0.345776000	2.681895000

8	2.429874000	0.277746000	3.907571000

Au_22_-HPO_4_^2-^

79	2.883800000	3.329882000	-2.354604000

79	1.441980000	0.832447000	-2.354648000

79	1.441880000	4.162334000	-0.000082000

79	0.000035000	1.664906000	-0.000026000

79	-1.441880000	-0.832469000	-0.000028000

79	4.325620000	0.832487000	-2.354595000

79	7.209388000	0.832435000	-2.354618000

79	5.767489000	-1.664943000	-2.354604000

79	5.767485000	3.329879000	-2.354614000

79	4.325588000	4.162309000	-0.000004000

79	5.767501000	1.664906000	-0.000002000

79	4.325540000	-4.162313000	-2.354641000

79	2.883645000	-1.664369000	-2.354876000

79	1.442071000	-0.832906000	0.000270000

79	4.325599000	-0.832463000	0.000071000

79	2.883794000	-3.329912000	0.000065000

79	2.883709000	1.664932000	0.000015000

79	1.441921000	-5.827284000	0.000045000

79	0.000011000	-3.329901000	-0.000040000

79	7.209396000	-0.832501000	0.000058000

79	4.325620000	-5.827325000	0.000010000

79	5.767507000	-3.329931000	0.000048000

15	2.155029000	0.508645000	2.927128000

8	1.930533000	1.550181000	3.967929000

8	2.451161000	-0.936344000	3.672934000

1	2.678620000	-1.582766000	2.991036000

8	0.923112000	0.222394000	1.985303000

8	3.422212000	0.690193000	2.002711000

Au_22_-Citrate^2-^

79	2.883869000	3.329930000	-2.354633000

79	1.441914000	0.832459000	-2.354627000

79	1.441892000	4.162315000	-0.000024000

79	-0.000010000	1.664912000	-0.000103000

79	-1.441913000	-0.832486000	0.000052000

79	4.325914000	0.832555000	-2.354579000

79	7.209512000	0.832645000	-2.354600000

79	5.766347000	-1.665528000	-2.355054000

79	5.767525000	3.329877000	-2.354573000

79	4.325579000	4.162285000	0.000016000

79	5.767337000	1.664876000	0.000130000

79	4.325749000	-4.162190000	-2.354654000

79	2.883841000	-1.664646000	-2.354743000

79	1.441884000	-0.832598000	-0.000048000

79	4.326630000	-0.831992000	0.000431000

79	2.883786000	-3.329999000	-0.000009000

79	2.883400000	1.664615000	-0.000015000

79	1.441881000	-5.827316000	0.000013000

79	-0.000032000	-3.329905000	-0.000054000

79	7.209451000	-0.832472000	0.000063000

79	4.325604000	-5.827312000	-0.000019000

79	5.767539000	-3.329822000	0.000233000

6	1.526389000	0.997410000	2.878498000

8	2.224774000	1.896733000	2.338714000

8	1.103287000	-0.061668000	2.352039000

6	1.138159000	1.240013000	4.346066000

1	1.126203000	0.283321000	4.869199000

1	1.898115000	1.894667000	4.789318000

6	-0.232694000	1.909253000	4.560645000

6	-0.276975000	3.350746000	4.013386000

1	-1.209951000	3.796574000	4.378900000

1	0.552977000	3.919011000	4.445727000

6	-1.404927000	1.057777000	4.040688000

1	-2.289212000	1.680662000	3.902628000

1	-1.135489000	0.619100000	3.075104000

6	-1.803411000	-0.045842000	4.984443000

6	-0.234530000	3.544454000	2.495406000

8	0.391506000	4.541019000	2.060530000

8	-2.903781000	-0.194028000	5.464710000

8	-0.814539000	-0.929059000	5.229570000

8	-0.890458000	2.704813000	1.813775000

8	-0.453440000	2.008418000	5.976960000

1	0.327820000	2.435343000	6.348103000

1	-1.203419000	-1.602244000	5.806265000

**References**

(1) da Costa, L. M.; Stoyanov, S. R.; Gusarov, S.; Tan, X.; Gray, M. R.; Stryker, J. M.; Tykwinski, R.; de M. Carneiro, J. W.; Seidl, P. R.; Kovalenko, A. Density functional theory investigation of the contributions of π–π stacking and hydrogen-bonding interactions to the aggregation of model asphaltene compounds. *Energy & Fuels* **2012**, *26* (5), 2727-2735, <https://doi.org/10.1021/ef202010p>.

(2) Frisch, M.; Trucks, G.; Schlegel, H.; Scuseria, G.; Robb, M.; Cheeseman, J.; Scalmani, G.; Barone, V.; Petersson, G.; Nakatsuji, H. Gaussian 16 vC. 01 (Revision C. 01). Wallingford CT: Gaussian. *Inc.[Google Scholar]* **2019**.

(3) Zhao, Y.; Truhlar, D. G. The M06 suite of density functionals for main group thermochemistry, thermochemical kinetics, noncovalent interactions, excited states, and transition elements: two new functionals and systematic testing of four M06-class functionals and 12 other functionals. *Theoretical chemistry accounts* **2008**, *120* (1), 215-241, <https://doi.org/10.1007/s00214-007-0310-x>.

(4) Hay, P. J.; Wadt, W. R. Ab initio effective core potentials for molecular calculations. Potentials for K to Au including the outermost core orbitals. *The Journal of chemical physics* **1985**, *82* (1), 299-310.

(5) Zossimova, E.; Fiedler, J.; Vollmer, F.; Walter, M. Hybrid quantum-classical polarizability model for single molecule biosensing. *Nanoscale* **2024**, *16* (11), 5820-5828, 10.1039/D3NR05396B, <https://doi.org/10.1039/D3NR05396B>.

(6) Vollmer, F.; Arnold, S. Whispering-gallery-mode biosensing: label-free detection down to single molecules. *Nature Methods* **2008**, *5* (7), 591-596, <https://doi.org/10.1038/nmeth.1221>.

(7) Arnold, S.; Khoshsima, M.; Teraoka, I.; Holler, S.; Vollmer, F. Shift of whispering-gallery modes in microspheres by protein adsorption. *Opt. Lett.* **2003**, *28* (4), 272-274, <https://doi.org/10.1364/OL.28.000272>.

(8) Vollmer, F.; Braun, D.; Libchaber, A.; Khoshsima, M.; Teraoka, I.; Arnold, S. Protein detection by optical shift of a resonant microcavity. *Applied Physics Letters* **2002**, *80* (21), 4057-4059, <https://doi.org/10.1063/1.1482797>.

(9) Toropov, N. A.; Houghton, M. C.; Yu, D.; Vollmer, F. Thermo-Optoplasmonic Single-Molecule Sensing on Optical Microcavities. *ACS Nano* **2024**, *18* (27), 17534-17546, <https://doi.org/10.1021/acsnano.4c00877>.

(10) Houghton, M. C.; Toropov, N. A.; Yu, D.; Bagby, S.; Vollmer, F. Single Molecule Thermodynamic Penalties Applied to Enzymes by Whispering Gallery Mode Biosensors. *Advanced Science* **2024**, *11* (35), 2403195, <https://doi.org/https://doi.org/10.1002/advs.202403195>.
